# Continuous non-adherent culture promotes transdifferentiation of human adipose-derived stem cells into retinal lineage

**DOI:** 10.1515/biol-2022-0760

**Published:** 2023-11-23

**Authors:** Qiying Ling, Jia-Jian Liang, Shaowan Chen, Chong-Bo Chen, Tsz Kin Ng, Yuqiang Huang

**Affiliations:** Joint Shantou International Eye Center of Shantou University and the Chinese University of Hong Kong, North Dongxia Road, Shantou, Guangdong, China; Department of Ophthalmology and Visual Sciences, The Chinese University of Hong Kong, Hong Kong, China

**Keywords:** adipose-derived stem cells, retinal cells, transdifferentiation, noggin/Dkk-1/IGF-1, non-adherent culture

## Abstract

Non-adherent culture is critical for the transdifferentiation of stem cells from mesoderm to neuroectoderm. Sphere culture has been reported to directly induce the adipose tissue cells to neural stem cells. Here we aimed to evaluate continuous non-adherent culture on the transdifferentiation potential of human adipose-derived stem cells (ASCs) into retinal lineage. Human ASCs were induced into retinal lineage by the treatment of noggin, dickkopf-related protein 1, and IGF-1 (NDI) under adherent and non-adherent culture. The NDI induction treatment with the adherent culture for 21 days promoted robust expression of retinal markers in the induced ASCs as compared to those without NDI induction on the adherent culture. With continuous non-adherent culture for 21 days, human ASCs could highly express retinal marker genes even without NDI induction treatment as compared to those on the adherent culture. The combination of continuous non-adherent culture with the NDI induction did not show a significant upregulation of the retinal marker expression as compared to either NDI induction with the adherent culture or continuous non-adherent culture without NDI induction treatment. In summary, both non-adherent culture and NDI induction medium could independently promote the transdifferentiation of human ASCs into retinal lineage. Yet, their combination did not produce an enhancement effect.

## 1 Introduction

Retinal diseases, including glaucoma, age-related macular degeneration, and diabetic retinopathy, are the leading causes of irreversible blindness and visual impairment, affecting more than 300 million individuals worldwide [1]. Retinal cell death is the pathological cause of vision loss among different retinal diseases. Human retina has limited intrinsic regenerative capacity, and currently there is still no effective clinical treatment to rescue the retinal cell loss. Stem cell therapy has been proposed as an emerging option for retinal disease treatment [2,3,4]. Stem cell replacement therapy for retinal diseases relies on the synthesis of retinal cells from stem cells to replace the diseased/damaged cells in the retina. Theoretically, retinal progenitor cells (RPCs) are the best source of stem cells for retinal cell differentiation because of the same lineage differentiation [5]; yet, it is not ethical to collect RPCs from the retina of a live patient with retinal disease as this would cause damage to the retina. Thus, extra-ocular stem cells are considered as the potential alternative sources for retinal cell synthesis. Protocols have been developed to induce human embryonic stem cells (ESCs) [6] and induced pluripotent stem cells (iPSCs) [7] into retinal lineage by inhibiting BMP and Wnt, and activating IGF-1 signaling pathways. Notably, we previously demonstrated that periodontal ligament-derived stem cells (PDLSCs) [8] and adipose-derived stem cells (ASCs) [9] can also be induced into retinal lineage by the treatment of noggin, dickkopf-related protein 1 (Dkk-1), and IGF-1 (NDI) similarly as ESCs. Apart from the NDI approach, additional strategies of transdifferentiation from PDLSCs/ASCs to retinal cells are worth investigating.

Non-adherent culture is critical for the transdifferentiation of stem cells from mesoderm to neuroectoderm. Previous study has adopted sphere culture to induce the cells from adipose tissue directly to neural stem cells [10]. Additionally, organoid culture is also applied to convert iPSCs into the laminar retina [11]. Herein we hypothesized that non-adherent culture could facilitate the transdifferentiation of ASCs into retinal lineage. Based on our previous investigation on the retinal differentiation of ASCs [9], we, in this study, aimed to determine the transdifferentiation potential of ASCs into retinal lineage, including RPCs, retinal ganglion cells (RGCs), and photoreceptors, with continuous non-adherent culture and in combination with the NDI induction strategy.

## 2 Methods

### 2.1 Human ASC culture

Four human ASC lines from abdominal fat have been previously established with characterization [9,12]. ASCs were cultured in Gibco Dulbecco’s Modified Eagle Medium (DMEM; Thermo Fisher Scientific, Waltham, MA) supplemented with 10% fetal bovine serum (FBS; Thermo Fisher Scientific) and 1% penicillin/streptomycin (P/S; Thermo Fisher Scientific). The medium was replaced every 3 days. Human ASCs at passage 3–5 were applied in the induction experiments.


**Informed consent:** Informed consent has been obtained from all individuals included in this study.
**Ethical approval:** The research related to human use has been complied with all the relevant national regulations, institutional policies, and in accordance with the tenets of the Helsinki Declaration, and has been approved by the Ethics Committee for Human Medical Research of Joint Shantou International Eye Center of Shantou University and The Chinese University of Hong Kong (approval number: EC20181106(6)-P01).

### 2.2 Retinal induction treatment on human ASCs

**Figure 1:**
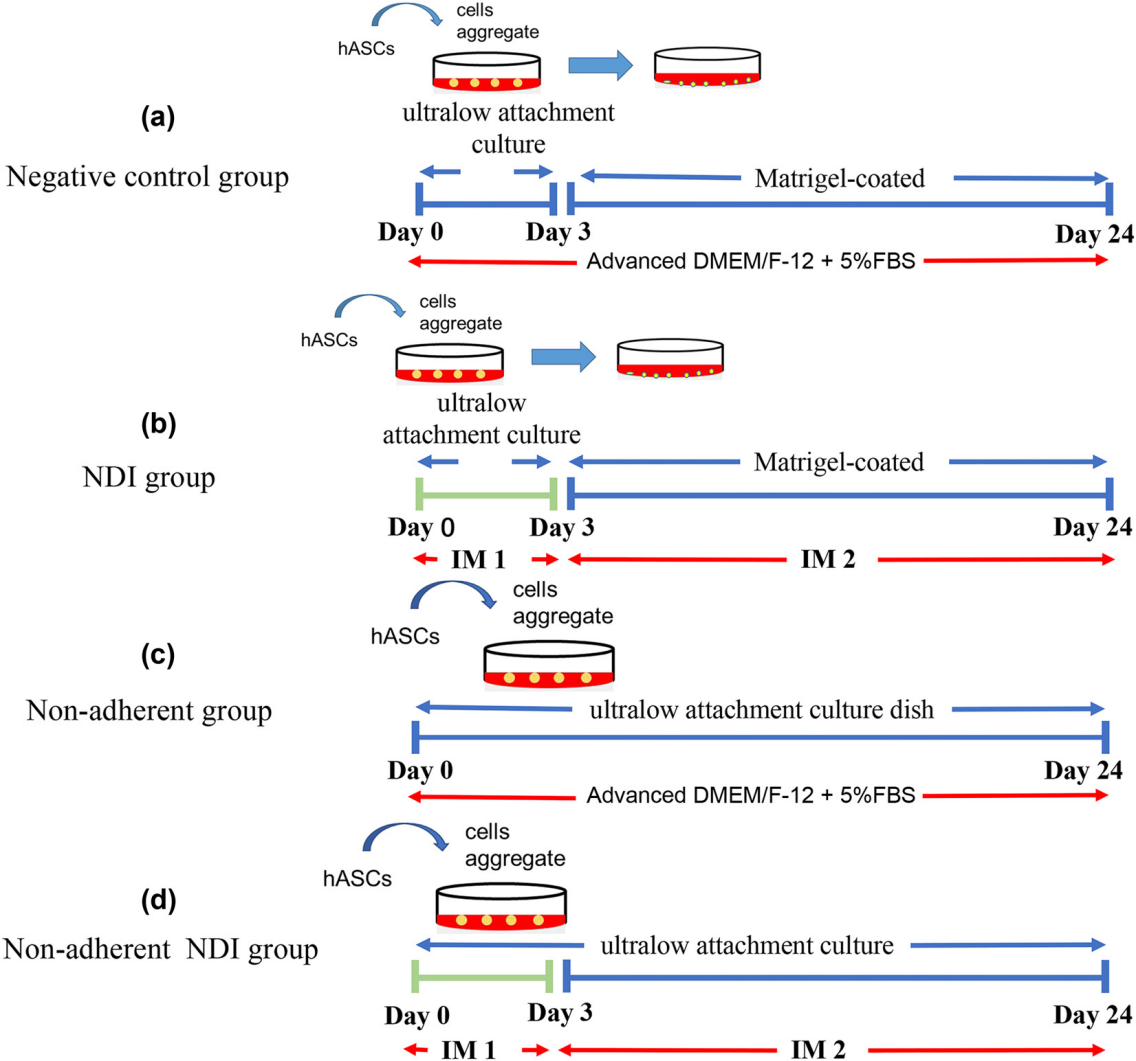
Schematic diagram of retinal induction treatment strategies on human ASCs. Human ASCs were treated in four approaches for 24 days: (a) Negative control group; (b) NDI group; (c) non-adherent group; and (d) non-adherent NDI group. IM1: Advanced DMEM/F-12 medium supplemented with 10% knockout serum replacement, 1× B27 supplement, 1 ng/mL noggin, 1 ng/mL Dkk-1, and 5 ng/mL IGF-1; IM2: Advanced DMEM/F-12 medium supplemented with 1× B27 supplement, 1× N2 supplement, 100 ng/mL noggin, 10 ng/mL Dkk-1, 100 ng/mL IGF-1, and 50 ng/mL bFGF; Medium in a and c groups: Advanced DMEM/F-12 medium supplemented with 5% FBS.

Human ASCs (initial seeding 1 × 10^6^ cells per dish) were treated in 4 approaches with different adherent and non-adherent culture conditions based on our previous established protocols (treatment first in non-adherent culture for 3 days and then followed by the adherent culture for 21 days) [8,9]: Group 1, the negative control group: 3-day non-adherent culture in the ultra-low attachment culture dish (Corning Life Sciences, Corning, NY) and 21-day adherent culture in Matrigel (Corning Life Sciences)-coated culture dish both treated with advanced DMEM/F-12 medium (Thermo Fisher Scientific) supplemented with 5% FBS and 1× P/S (Figure 1a); Group 2, the NDI group: 3-day non-adherent culture in the ultra-low attachment culture dish treated with the induction medium 1 (IM1; advanced DMEM/F-12 medium supplemented with 10% knockout serum replacement (Thermo Fisher Scientific), 1× B27 supplement (Thermo Fisher Scientific), 1 ng/mL noggin (PeproTech), 1 ng/mL dickkopf-related protein 1 (Dkk-1; PeproTech), 5 ng/mL IGF-1 (PeproTech), and 1× P/S) and 21-day adherent culture in Matrigel-coated culture dish treated with the induction medium 2 (IM2; advanced DMEM/F-12 medium supplemented with 1× B27 supplement, 1× N2 supplement (Thermo Fisher Scientific), 100 ng/mL noggin, 10 ng/mL Dkk-1, 100 ng/mL IGF-1, 50 ng/mL basic fibroblast growth factor (bFGF; PeproTech), and 1× P/S) (Figure 1b); Group 3, non-adherent group: 3-day non-adherent culture and 21-day continuous non-adherent culture in the ultra-low attachment culture dish treated with advanced DMEM/F-12 medium supplemented with 5% FBS and 1× P/S (Figure 1c); Group 4, non-adherent NDI group: non-adherent culture in the ultra-low attachment culture dish treated with 3-day IM1 and 21-day IM2 (Figure 1d). The treated cells were collected before treatment and at every 3 days for further gene expression analysis.

### 2.3 Gene expression analysis

Total RNA was extracted and purified with the TRIzol reagent (Thermo Fisher Scientific) according to the manufacturer’s protocol. Total RNA (1 μg) was reverse transcribed into complementary DNA with PrimeScript RT reagent Kit (TaKaRa Bio Inc., Shiga, Japan). The expressions of RPCs (*PAX6, VSX2, LHX2, SOX2, RAX,* and *NES*), RGCs (*ATOH7* and *TUBB3*), photoreceptor (*CRX, NRL, RHO,* and *RCVRN*), and stem cell (*KIT*) marker genes were amplified by the SYBR Green I Master Mix (TaKaRa Bio Inc.) with respective primers (Table S1) in LightCycler 480 system (Roche, Basel, Switzerland) according to our previously established protocol [13]. The housekeeping gene (*ACTB*) was used for normalization. Relative gene expression was calculated by the 2^−ΔΔCt^ method. The specificity of the polymerase chain reaction for each gene in the gene expression analysis was confirmed by the melting curve analysis (Figure S1).

### 2.4 Statistical analysis

Data were presented as the mean values of the results from 4 ASC lines ± standard deviation (SD). One-way analysis of variance (ANOVA) with post-hoc Tukey test was applied to compare the data among different groups. All statistical analyses were performed using commercially available software (IBM SPSS Statistics 21; SPSS Inc., Chicago, IL). Statistical significance was defined as *p* < 0.05.

## 3 Results

### 3.1 Morphology of human ASCs along the retinal induction treatment

**Figure 2:**
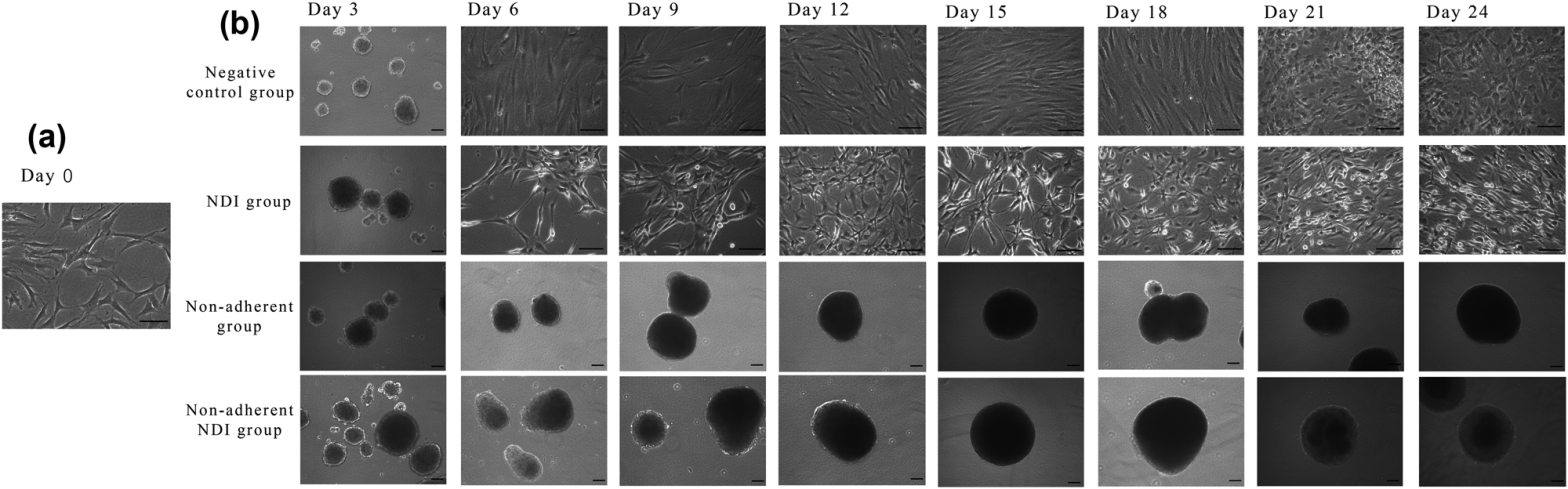
Morphology of human ASCs after different retinal induction treatment approaches. Human ASCs were treated in four approaches for 24 days, and the images were taken every 3 days. (a) Before the retinal induction treatment, human ASCs were in fibroblast-like shape. (b) The negative control group and the NDI group were cultured on the Matrigel-coated dishes, while the non-adherent group and the non-adherent NDI group were cultured in the ultra-low attachment dishes without Matrigel. In the ultra-low attachment dishes, human ASCs formed spheres. For the NDI group in the Matrigel-coated dishes, the induced human ASCs gradually acquired the neuronal cell-like shape and formed neural-network-like morphology. Human ASCs in the negative control group without NDI treatment returned back to the fibroblast-like shape along the 21 days adherent culture. Scale bar: 100 μm.

Before the retinal induction treatment, human ASCs were in fibroblast-like shape (Figure 2a). For the first 3 days treatment in the ultra-low attachment dishes, human ASCs formed spheres in all treatment groups (Figure 2b). For the treatment groups with continuous non-adherent culture, the induced ASCs were maintained in the sphere form, confirming the non-adherent culture condition. In contrast, ASCs cultured in the Matrigel-coated dishes gradually acquired the neuronal cell-like shape and formed neural-network-like morphology under the NDI treatment. The ASCs in the negative control group without NDI treatment returned back to the fibroblast-like shape and continued rapid proliferation along the 21-day adherent culture.

### 3.2 Retinal transdifferentiation of human ASCs under NDI treatment

**Figure 3:**
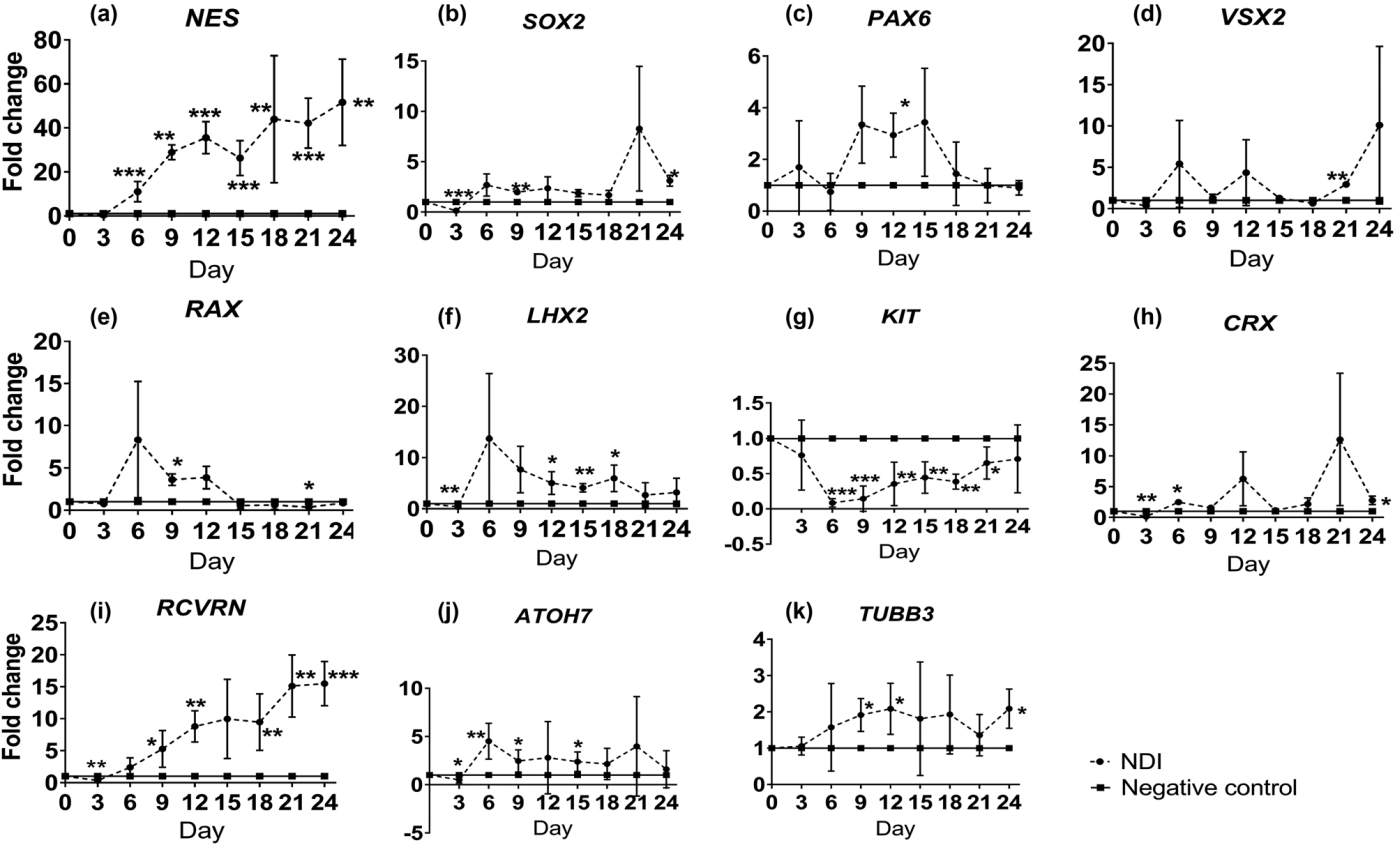
Comparison of retinal marker expression in human ASCs with or without NDI treatment. Human ASCs were treated in the ultra-low attachment culture dish for 3 days and in Matrigel-coated culture dish for 21 days with or without NDI induction medium. The expression of retinal marker genes in human ASCs with NDI treatment was evaluated by SYBR Green PCR every 3 days: (a) *NES*, (b) *SOX2*, (c) *PAX6*, (d) *VSX2*, (e) *RAX*, (f) *LHX2*, (g) *KIT*, (h) *CRX*, (i) *RCVRN*, (j) *ATOH7*, and (k) *TUBB3*. The data were presented as mean values ± SD and compared with the one-way ANOVA for all the four treatment groups followed by the pairwise post-hoc Tukey test. *P < 0.05, **P < 0.01, ***P < 0.001 compared to the negative control group.

To confirm the retinal transdifferentiation ability of human ASCs, the NDI induction treatment was adopted as a positive control. Gene expression analysis demonstrated that RPC marker *NES* gene was significantly and gradually upregulated in human ASCs along the NDI treatment under adherent culture from Day 6 by 11.05 ± 2.30 folds (*P* < 0.01) to Day 24 by 51.66 ± 9.83 folds (*P* < 0.01) as compared to the negative control group (Figure 3a). Similarly, the expressions of other RPC markers, *SOX2* (3.12 ± 0.31 folds, *P* < 0.05 at Day 24; Figure 3b)*, PAX6* (2.94 ± 0.49 folds, *P* < 0.05 at Day 12; Figure 3c)*, VSX2* (2.94 ± 0.14 folds, *P* < 0.01 at Day 21; Figure 3d), *RAX* (3.63 ± 0.40 folds, *P* < 0.05 at Day 9; Figure 3e), and *LHX2* (5.97 ± 1.51 folds, *P* < 0.05 at Day 18; Figure 3f), were also significantly increased as compared to the negative control group. Meanwhile, the expression of stem cell marker *KIT* was significantly downregulated from Day 6 by 22.02 ± 18.85 folds (*P* < 0.001) to Day 21 by 1.71 ± 0.59 folds (*P* < 0.05) as compared to the negative control group (Figure 3g). Moreover, the expressions of photoreceptor markers *CRX* and *RCVRN* were significantly upregulated by 2.49 ± 0.25 folds at Day 6 (*P* < 0.05; Figure 3h) and 15.49 ± 1.73 folds at Day 24 (*P* < 0.001; Figure 3i), whereas that of RGC markers *ATOH7* and *TUBB3* were significantly upregulated by 4.52 ± 0.93 folds at Day 6 (*P* < 0.01; Figure 3j) and 2.09 ± 0.27 folds at Day 24 (*P* < 0.05; Figure 3k), respectively. Collectively, our results confirmed that NDI treatment could promote the transdifferentiation of human ASCs into retinal lineage.

### 3.3 Retinal transdifferentiation of human ASCs under non-adherent culture

**Figure 4:**
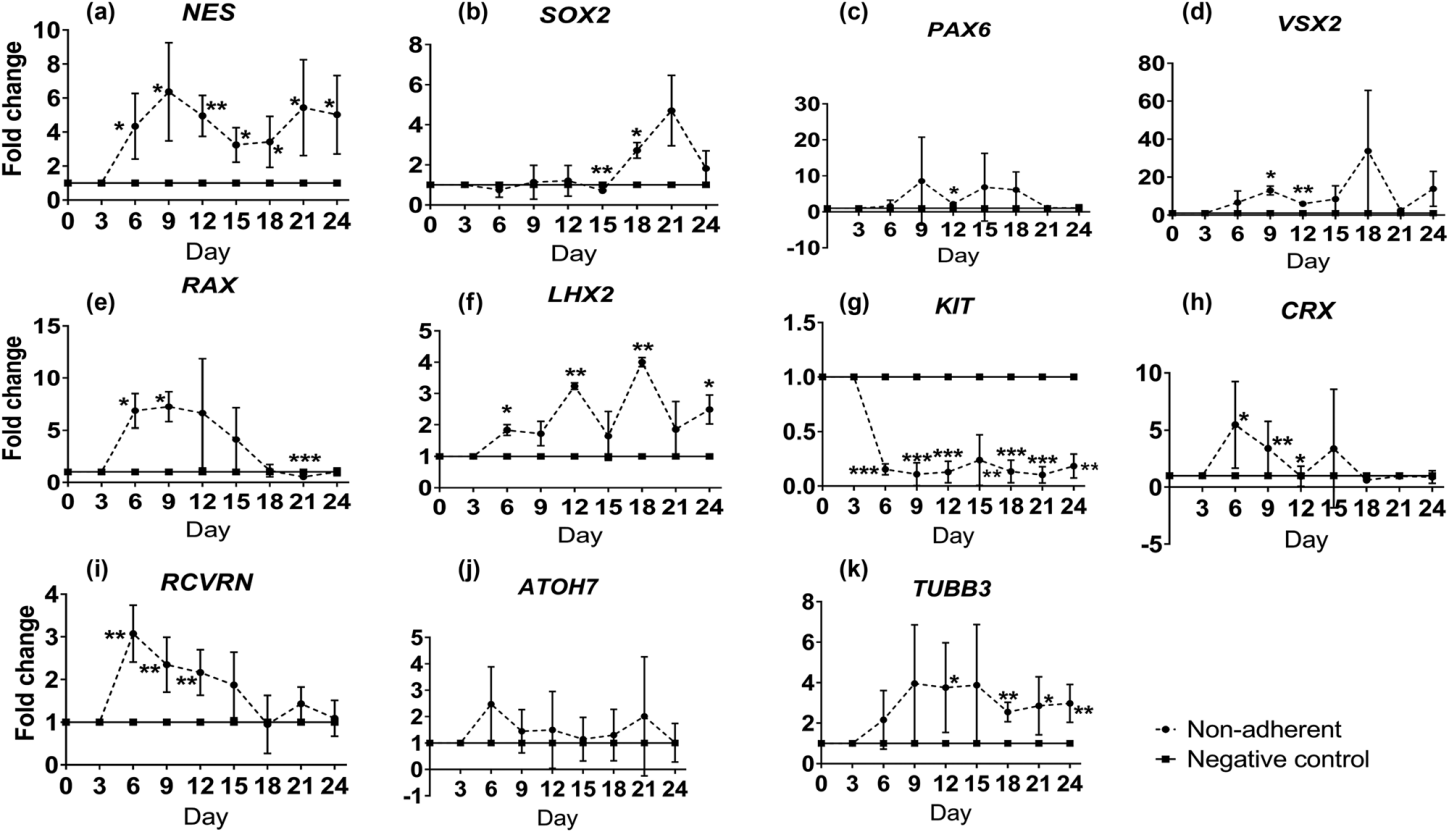
Comparison of retinal marker expression in human ASCs with continuous non-adherent culture to the adherent culture. Human ASCs were treated in the ultra-low attachment culture dish for 24 days or in the ultra-low attachment culture dish for 3 days and in Matrigel-coated culture dish for 21 days without NDI induction medium. The expression of retinal marker genes in human ASCs under non-adherent culture was evaluated by SYBR Green PCR every 3 days: (a) *NES*, (b) *SOX2*, (c) *PAX6*, (d) *VSX2*, (e) *RAX*, (f) *LHX2*, (g) *KIT*, (h) *CRX*, (i) *RCVRN*, (j) *ATOH7*, and (k) *TUBB3*. The data were presented as mean values ± SD and compared with the one-way ANOVA for all the four treatment groups followed by the pairwise post-hoc Tukey test. *P < 0.05, **P < 0.01, ***P < 0.001 compared to the negative control group.

To evaluate the retinal transdifferentiation ability of non-adherent culture, the expression of retinal gene markers in human ASCs without NDI under non-adherent culture was compared to the negative control group under adherent culture. Similar to the NDI treatment, significant upregulation of RPC marker *NES* gene was observed in human ASCs under non-adherent culture since Day 6 by 4.34 ± 0.97 folds (*P* < 0.05) and maintained steadily to Day 24 by 5.02 ± 1.15 folds (*P* < 0.01) as compared to the negative control group (Figure 4a). Other RPC markers, *SOX2* (2.73 ± 0.23 folds, *P* < 0.05 at Day 18; Figure 4b)*, PAX6* (2.17 ± 0.35 folds, *P* < 0.05 at Day 12; Figure 4c)*, VSX2* (12.95 ± 1.35 folds, *P* < 0.05 at Day 9; Figure 4d)*, RAX* (7.25 ± 0.83 folds, *P* < 0.05 at Day 9; Figure 4e), and *LHX2* (4.00 ± 0.08 folds, *P* < 0.01 at Day 18; Figure 4f), were also significantly increased as compared to the negative control group. Consistently, the expression of stem cell marker *KIT* was significantly downregulated from Day 6 by 7.06 ± 2.10 folds (*P* < 0.001) to Day 24 by 6.80 ± 2.84 folds (*P* < 0.01) as compared to the negative control group (Figure 4g). Additionally, the expressions of photoreceptor markers *CRX* and *RCVRN* were significantly upregulated by 7.47 ± 0.85 folds at Day 6 (*P* < 0.05; Figure 4h) and 2.16 ± 0.27 folds at Day 12 (*P* < 0.01; Figure 4i), respectively. For the RGC markers, only *TUBB3* was significantly upregulated in human ASCs under non-adherent culture by 2.97 ± 0.47 folds at Day 24 (*P* < 0.001; Figure 4k). Collectively, our results demonstrated that human ASCs would lose the stem cell identity under the non-adherent culture and could transdifferentiate into retinal lineage.

### 3.4 Retinal transdifferentiation of human ASCs under non-adherent culture with NDI treatment

**Figure 5:**
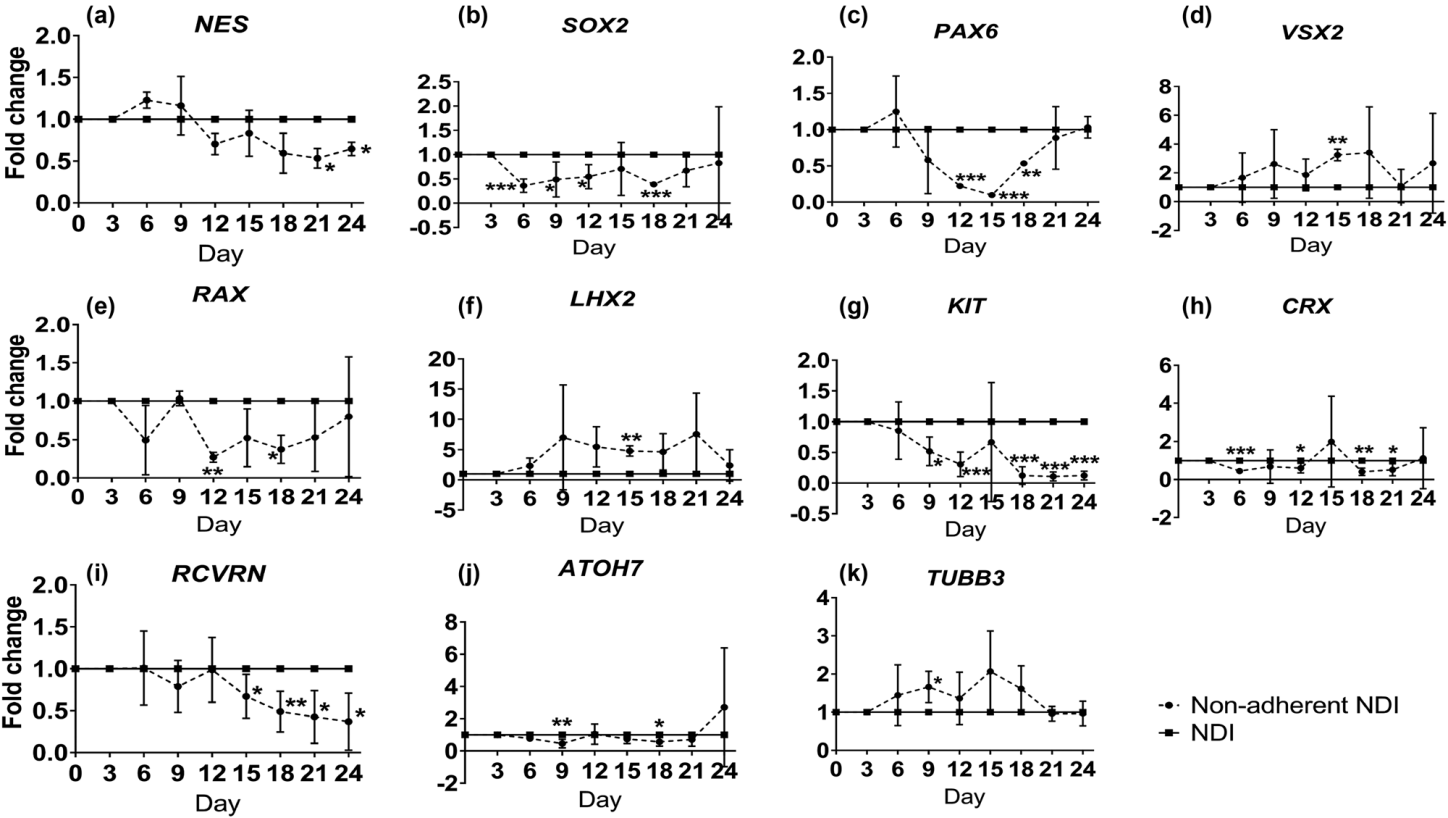
Comparison of retinal marker expression in human ASCs with the NDI treatment in adherent or continuous non-adherent culture. Human ASCs were treated with NDI induction medium in the ultra-low attachment culture dish for 24 days or in the ultra-low attachment culture dish for 3 days and in Matrigel-coated culture dish for 21 days. The expression of retinal marker genes in human ASCs under non-adherent culture with NDI treatment was evaluated by SYBR Green PCR every 3 days: (a) *NES*, (b) *SOX2*, (c) *PAX6*, (d) *VSX2*, (e) *RAX*, (f) *LHX2*, (g) *KIT*, (h) *CRX*, (i) *RCVRN*, (j) *ATOH7*, and (k) *TUBB3*. The data were presented as mean values ± SD and compared with the one-way ANOVA for all the four treatment groups followed by the pairwise post-hoc Tukey test. *P < 0.05, **P < 0.01, ***P < 0.001 compared to those with the NDI treatment in adherent culture.

We further tested the NDI induction treatment with the non-adherent culture. The expressions of *NES* (0.65 ± 0.05 folds, *P* < 0.05 at Day 24; Figure 5a), *SOX2* (0.54 ± 0.12 folds, *P* < 0.05 at Day 12; Figure 5b)*, PAX6* (0.53 ± 0.01 folds, *P* < 0.01 at Day 18; Figure 5c)*, RAX* (0.37 ± 0.11 folds, *P* < 0.05 at Day 18; Figure 5e), and *KIT* (0.13 ± 0.04 folds, *P* < 0.001 at Day 24; Figure 5g) in human ASCs with the NDI treatment under the non-adherent culture were found to be significantly lower than that under the adherent culture. Moreover, significantly lower expressions of *CRX* (0.60 ± 0.13 folds, *P* < 0.05 at Day 12; Figure 5h), *RCVRN* (0.67 ± 0.13 folds, *P* < 0.05 at Day 15; Figure 5i), and *ATOH7* (0.57 ± 0.14 folds, *P* < 0.05 at Day 18; Figure 5j) were also found in human ASCs with the NDI treatment under the non-adherent culture as compared to that under the adherent culture. In contrast, significantly higher expressions of *VSX2* (3.25 ± 0.23 folds, *P* < 0.01 at Day 15; Figure 5d), *LHX2* (4.81 ± 0.50 folds, *P* < 0.01 at Day 15; Figure 5f), and *TUBB3* (1.66 ± 0.24 folds, *P* < 0.05 at Day 9; Figure 5k) were found in human ASCs with the NDI treatment under the non-adherent culture as compared to that under the adherent culture.

**Figure 6:**
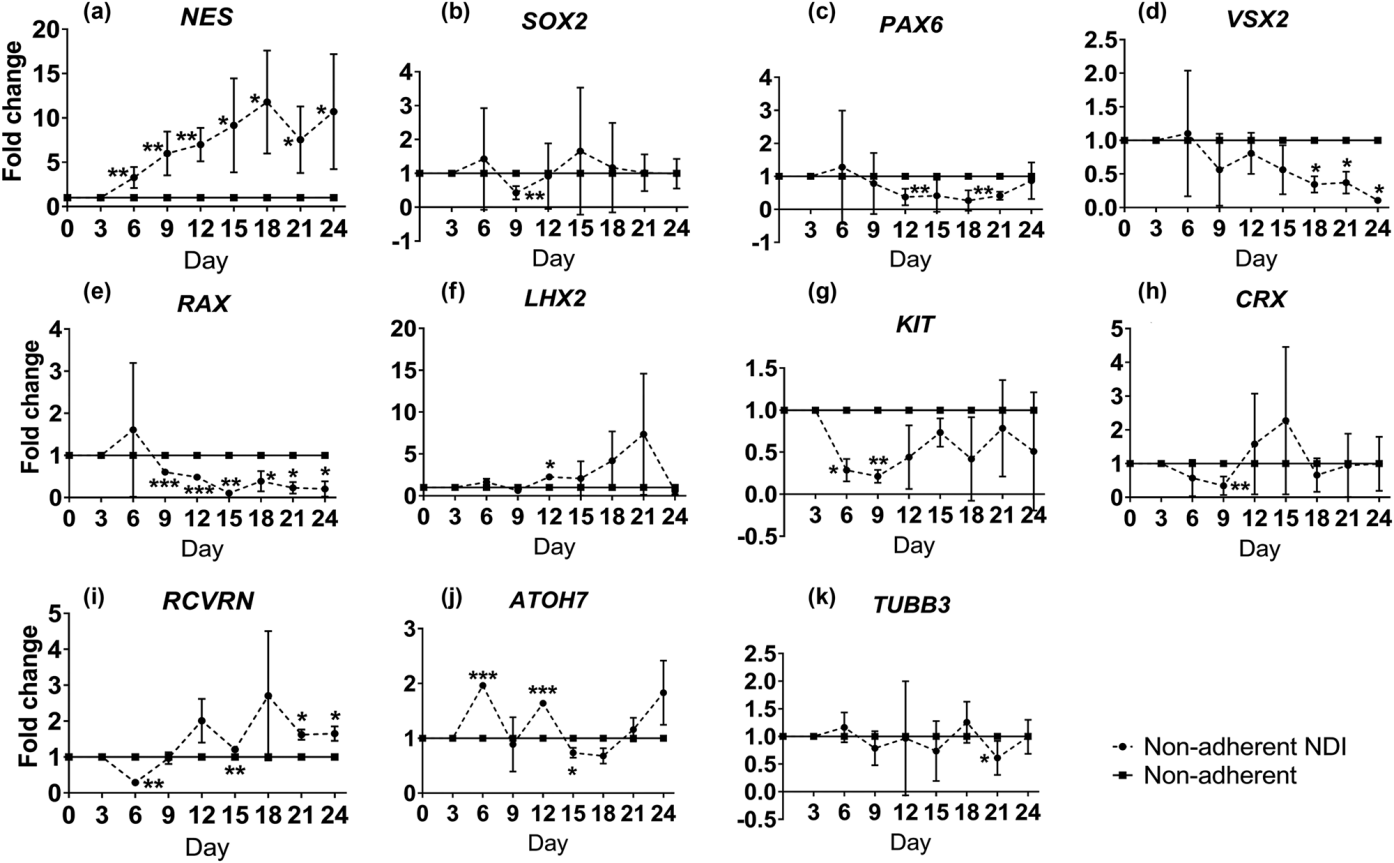
Comparison of retinal marker expression in human ASCs under continuous non-adherent culture with or without NDI treatment. Human ASCs were treated in the ultra-low attachment culture dish for 24 days with or without NDI induction medium. The expression of retinal marker genes in human ASCs under non-adherent culture with NDI treatment was evaluated by SYBR Green PCR every 3 days: (a) *NES*, (b) *SOX2*, (c) *PAX6*, (d) *VSX2*, (e) *RAX*, (f) *LHX2*, (g) *KIT*, (h) *CRX*, (i) *RCVRN*, (j) *ATOH7*, and (k) *TUBB3*. The data were presented as mean values ± SD and compared with the one-way ANOVA for all the four treatment groups followed by the pairwise post-hoc Tukey test. *P < 0.05, **P < 0.01, ***P < 0.001 compared to those under non-adherent culture without NDI treatment.

Compared to the non-adherent culture without NDI treatment, significantly higher expressions of *NES* (2.25 ± 0.19 folds, *P* < 0.05 at Day 12; Figure 6a), *LHX2* (11.78 ± 2.90 folds, *P* < 0.05 at Day 18; Figure 6f), *RCVRN* (1.65 ± 0.12 folds, *P* < 0.05 at Day 24; Figure 6i), and *ATOH7* (1.96 ± 0.02 folds, *P* < 0.001 at Day 6; Figure 6j) were found in human ASCs with the NDI treatment under the non-adherent culture. Yet, significantly lower expression of *SOX2* (0.43 ± 0.10 folds, *P* < 0.01 at Day 9; Figure 6b), *PAX6* (0.38 ± 0.13 folds, *P* < 0.01 at Day 12; Figure 6c), *VSX2* (0.37 ± 0.09 folds, *P* < 0.05 at Day 21; Figure 6d), *RAX* (0.60 ± 0.01 folds, *P* < 0.001 at Day 9; Figure 6e), *KIT* (0.20 ± 0.04 folds, *P* < 0.01 at Day 9; Figure 6g), *CRX* (0.34 ± 0.14 folds, *P* < 0.01 at Day 9; Figure 6h), and *TUBB3* (0.61 ± 0.15 folds, *P* < 0.05 at Day 21; Figure 6k) were found in human ASCs with the NDI treatment under the non-adherent culture as compared to that without NDI treatment. Collectively, our results indicated that the combination of NDI treatment and non-adherent culture did not further enhance the transdifferentiation of human ASCs into retinal lineage as compared to the NDI treatment or non-adherent culture alone.

## 4 Discussion

Results from this study showed that: (1) the NDI treatment could promote the transdifferentiation of human ASCs into retinal lineage [9]; (2) non-adherent culture could promote the transdifferentiation of human ASCs into retinal lineage; (3) the combination of NDI treatment and non-adherent culture could not further enhance the transdifferentiation of human ASCs into retinal lineage as compared to either NDI treatment or non-adherent culture. Collectively, our results demonstrate the potential of non-adherent culture promoting the transdifferentiation of ASCs into retinal lineage.

Various signaling pathways are involved in the regulation of retinogenesis, including Wnt, TGF-β, and FGF [14,15]. In the *in vitro* culture system, inhibiting BMP and Wnt, as well as activating IGF-1 signaling pathways by the NDI treatment, has been applied to induce human ESCs and iPSCs into retinal lineage [6,7]. We previously also demonstrated that the NDI induction treatment is able to induce human PDLSCs and ASCs into retinal cells [8,9]. In this study, we confirmed that, upon NDI induction treatment, significant upregulation of RPC, photoreceptor, and RGC gene markers was found in the NDI-treated human ASCs as compared to the negative control group (Figure 3), indicating the successful transdifferentiation of human ASCs into retinal lineage. The genes selected in this study are based on our previous studies [6,7,8,9], and they are the specific marker genes for RPCs, RGCs, and photoreceptors.

Development of the retina and its cell types proceeds in a highly regulated manner [16]. The neural retina with six different types of retinal cells is generated from the inner layer of optic cup. Retinogenesis can also be resembled in the *in vitro* culture system, which could be spontaneously initiated in ESCs using the organoid culture system [17]. Non-adherent culture is the basis of the organoid culture [18]. Under the non-adherent culture system, neural stem cells have the ability to aggregate and proliferate in the form of neurospheres [19]. Non-adherent culture is also critical for the transdifferentiation of stem cells from mesoderm to neuroectoderm. A previous study has reported that sphere culture for 7 days could directly induce the cells from adipose tissue to neural stem cells [10]. Similarly, hanging drop culture for 2 days can also induce human ASCs into anterior neuroectoderm [20]. Consistently, we previously showed that the initial 3 days non-adherent culture is critical and essential for the transdifferentiation of human ASCs and PDLSCs into the retinal lineage [8,9]. A previous study also demonstrated that rat bone marrow stromal stem cells can differentiate into cells expressing nestin and retinal pigment epithelial markers by the neurosphere culture [21]. Yet, whether extending the time of non-adherent culture would be beneficial to the retinal transdifferentiation is still unknown. This study, for the first time, demonstrated that, even without the NDI induction medium, the continuous non-adherent culture (Figure 2) alone could upregulate the expression of retinal marker genes in the treated human ASCs as compared to the adherent culture (Figure 4). However, the combination of the NDI treatment and the non-adherent culture did not further enhance the expression of retinal marker genes as compared to the NDI treatment (Figure 5) or non-adherent culture alone (Figure 6). Additional functional assays and transplantation studies in disease models can help to evaluate the functions of the differentiated cells. In addition, further investigations are also needed to delineate the mechanisms on the combined treatment of NDI and the non-adherent culture in the retinal differentiation of human ASCs.

The non-adherent culture system itself possesses its limitations [22]. First, different cell densities could affect the microenvironment, thereby affecting the cell proliferation and differentiation. Second, the growth factor concentrations in the culture medium and the cell passages could lead to different characteristics of the neurospheres. This could be a reason why the NDI treatment would not be able to further enhance the retinal marker gene expression in human ASCs under the non-adherent culture, possibly due to the suboptimal growth factor concentrations. Third, although neurosphere culture is a specific characteristic of neural stem cells, retinal transdifferentiation of human ASCs under continuous non-adherent culture could not maintain the induced cells in the RPC stage and prevent the expression of mature retinal markers, which could explain the instable expression of RPC, RGC, and photoreceptor markers over times (Figures 3–6). Besides, different coating materials, scaffolds, and substrates can have different roles in the stem cell transdifferentiation. As RPCs or retinal precursor cells would be better than mature retinal cells in cell replacement therapy for retinal disease treatment [23], further studies are needed to control the transdifferentiation of human ASCs specifically to the stage of RPC.

## 5 Conclusion

This study revealed that non-adherent culture and NDI induction medium showed abilities to promote the transdifferentiation of human ASCs into retinal lineage. Yet, their combination did not produce an enhancement effect. Further optimization on the retinal transdifferentiation protocol could help to consolidate the application of ASCs for retinal disease treatment in future.

## Supplementary Material

Supplementary material
