# Supplementary material for: Continuous non-adherent culture promotes transdifferentiation of human adipose-derived stem cells into retinal lineage
Source: Open Life Sci. 2023 Nov 23;18(1):20220760. doi: 10.1515/biol-2022-0760 (PMC10668113; doi:10.1515/biol-2022-0760)
Supplement: Supplementary material [file biol-2022-0760-sm.pdf]

Supplementary material

Table S1: Primers used in the gene expression analysis

Gene	Sequence (5' > 3')	Tm (°C)	Product size (bp)	References
PAX6	F : AGAGGTCAGGCTTCGCTAATG	60	110	NM_000280.4
	R : TCAGATTCCTATGCTGATTGGTGA			
CRX	F : AGGGTTCAGGTTTGGTTCAAG	60	193	NM_000554.4
	R : GAGGGGGACTGTAGGAATCTG			
NRL	F : CATTGGGGCTGAGTCCTGAAGA	60	178	NM_006177.3
	R : TTTAGCTCCCGCACAGACATCG			
ATOH7	F : CAGACCTATGGACGCAATCA	60	105	NM_145178.3
	R : TTTTCACAGCAATCAACCCA			
LHX2	F : GAAGCAGCTCGCGCAAAAG	60	188	NM_004789.4
	R : TGAGCGAGGCGTTGGAGA			
RCVRN	F : GGAGATCGTCATGGCTATTTTCAA	60	165	NM_002903.2
	R : GCCAGTGTCCCCTCAATGAA			
NES	F : GATCGCTCAGGTCCTGGAAG	60	160	NM_006617.1
	R : CTTGGGGTCCTGAAAGCTGA			
TUBB3	F : GGGAGATCGTGCACATCCAG	60	180	NM_006086.3
	R : GAGGCACGTACTTGTGAGAAGA			
VSX2	F : CTTGCAGCCATTGGGCAGA	60	161	NM_182894.3
	R : AGGAGGTAAAGATTGTCCTGTGTC			
SOX2	F : GACAGTTACGCGCACATGAA	60	181	NM_003106.4
	R : TCATGTAGGTCTGCGAGCTG			
RAX	F : GCGTTCGAGAAGTCCCACTA	60	156	NM_013435.3
	R : CTGCAGCTTCATGGAGGACA			
KIT	F : AAACGCTCGACTACCTGTGAA	60	130	NM_000222
	R : ATAGGGGCTGCTTCCTAAAGAG			
ACTB	F : CCCTGGACTTCGAGCAAGAG	60	153	NM_001101.3
	R : ACTCCATGCCCAGGAAGGAA			

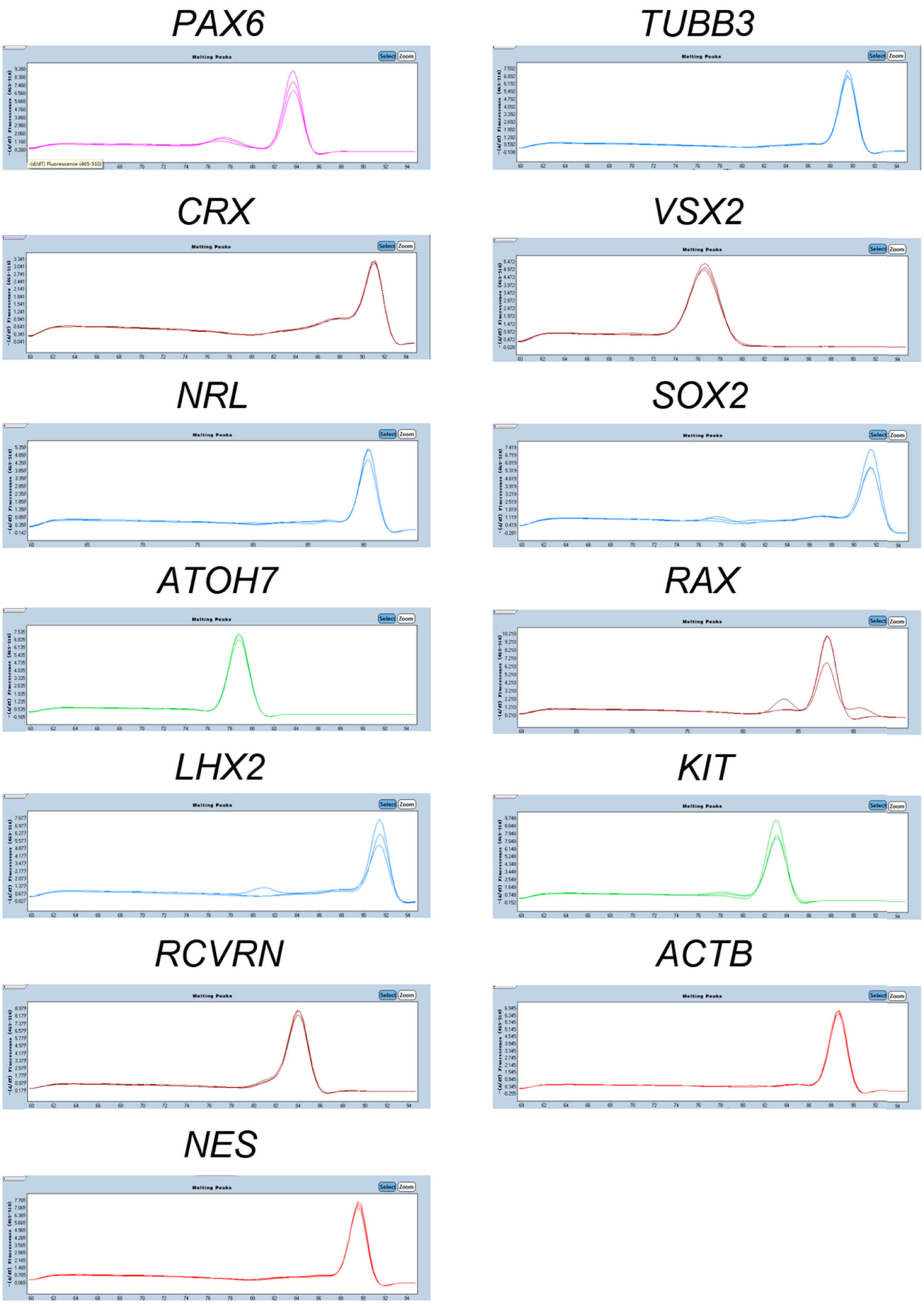

Figure S1: The melting curve analysis of each gene in the gene expression analysis.
